# Demographics, politics, and health factors predict mask wearing during the COVID-19 pandemic: a cross-sectional study

**DOI:** 10.1186/s12889-021-11424-1

**Published:** 2021-07-15

**Authors:** George B. Cunningham, Calvin Nite

**Affiliations:** Texas A&M University, College Station, USA

## Abstract

**Background:**

Wearing a protective face covering can reduce the spread of COVID-19, but Americans’ compliance with wearing a mask is uneven. The purpose of this study is to examine the association between health determinants (Health Behaviors, Clinical Care, Social and Economic Conditions, and the Physical Environment) and mask wearing at the county level.

**Methods:**

Data were collected from publicly available sources, including the County Health Rankings and the *New York Times*. The dependent variable was the percent of county residents who reported frequently or always wearing a mask when in public. County demographics and voting patterns served as controls. Two-levels random effects regression models were used to examine the study hypotheses.

**Results:**

Results indicate that, after considering the effects of the controls, Health Behaviors were positively associated with mask wearing, the Physical Environment held a negative association, and Clinical Care and Social and Behavioral Factors were unrelated.

**Conclusions:**

Results indicate that patterns of healthy behaviors can help predict compliance with public health mandates that can help reduce the spread of COVID-19. From an instutitional theory perspective, the data suggest counties develop collective values and norms around health. Thus, public health officials can seek to alter governance structures and normative behaviors to improve healthy behaviors.

## Introduction

As of January 2021, more than 1 in every 1000 US residents had died of COVID-19 (CDC, 2021). In addition to the stunning mortality rate were other health and psychological health morbidities [1, 2], increased death by suicide [3], and economic tolls [4]. As a result, public health experts encouraged people to employ a variety of safety measures, including wearing a mask that covers their mouth and nose [5]. Indeed, researchers have shown that protective face coverings can reduce the spread of COVID-19 [6, 7]. These benefits accrue even with minimally effective face coverings [8], and they are especially effective when coupled with other preventative behaviors, such as distancing from others [9]. As a result, many organizations – including the Centers for Disease Control and Prevention [10], American Hospital Association, American Medical Association, and American Nurses Association [11] – advocated for mask wearing as a way to prevent the spread of COVID-19.

Despite the scientific support for wearing masks, the public’s attitudes are equivocal. Part of the variability could be due to the uneven messaging from major health organizations, including the Centers for Disease Control and Prevention, and the World Health Organization [12]. Subsequent scholarship on the issue, though, has also pointed to other factors. Researchers out of Germany [13], for example, conducted a series of studies, finding even though mandatory mask requirements increased compliance, acceptance of the polices was only moderate. Further, participants stigmatized non-mask wearers. In an observational study in the US in June of 2020, just 41% of people wore a mask [14]. There were a number of factors associated with mask wearing, including older age, being female, and living in a suburban or urban setting [14]. Other researchers have shown that endorsement of masculinity [15, 16], and following a conservative political ideology [17] were linked with opposition to mask wearing. Collectively, this scholarship shows that a number of factors influence people’s decision to wear a mask during the COVID-19 pandemic.

In the current study, we expand this research in several ways. First, we extend the unit of analysis from the individual to the county in the United States. Drawing from an institutional theory perspective [18, 19], we argue that such an analysis allows for examination of how collective norms and practices within a community can influence health-related behaviors. Institutions and their engrained logics provide the foundational schema of proper, legitimate action within a given setting [20, 21]. Our focus here is geographic communities, specifically counties, wherein community membership is reflective of shared interests, resources, and understandings that shape action [22, 23]. These types of communities are influenced by local regulatory, social-normative, and cultural-cognitive institutions that all shape actions within the boundaries of the community [23]. Prior research of health behaviors has shown that, broadly speaking, cultural norms and institutions are indicative of the health of a society [24]. Thus, an institutional perspective likely provides insight into the manifestation of healthy behaviors, including the propensity to wear masks during the COVID-19 pandemic, within a given community.

As a second extension of previous research, we draw from Hood et al.’s [25] multi-level model, which shows how institutional theory might explain mask wearing [25].The authors developed a framework focusing on the ways in which modifiable factors collectively influenced quality of life and mortality. The most distal of the health determinants is the *physical environment*, including pollution, housing problems, and commute times. Hood et al. then specified *social and economic factors*, such as educational quality, employment, income, family and social support, and the safety of communities. Consistent with other entities, such as the World Health Organization, the authors noted that social and economic factors are likely to exert the strongest effects on health outcomes. *Clinical care*, including the access to and quality of care, represents the next health determinant. Finally, Hood et al. argued that *health behaviors* would influence quality of life and morbidity. Examples include tobacco use, diet and exercise, alcohol and drug use, and sexual activity. Drawing from a national sample of over 3000 US counties, Hood et al. found broad support for their model, as the factors were predictive of health outcomes, as measured by the length of life and quality of life among county residents [25]. Other researchers have also examined multilevel determinants of county-level health outcomes, largely observing a similar pattern [26–29].

Researchers employing the county health rankings model have largely focused on overall health outcomes. From an institutional theory perspective [23, 24], however, a county’s health behaviors, clinical care, social and economic factors, and physical environment, are also reflective of the norms, values, and culture surrounding health among community members. If this is the case, then the collective health factors are likely predictive of other health activities and behaviors. For example, researchers [30, 31] have adopted portions of Hood et al.’s model to show that a variety of county-level health factors were associated with COVID-19 mortality rates. Similarly, primary care physician rate (a factor of clinical care in Hood et al.’s model) is predictive of COVID-19 deaths at the county level [32].

Drawing from this collective work, we anticipated that the county-level health factors would be predictive of mask wearing among county residents. Consistent with our theoretical framework and Hood et al.’s model, we hypothesized that the health behaviors (Hypothesis 1), clinical care (Hypothesis 2), social and economic conditions (Hypothesis 3), and the physical environment (Hypothesis 4) would be positively associated with mask wearing among county residents, respectively.

## Method

### Study design, data sources, and variables

For this cross-sectional study, we collected data from counties or county equivalents (boroughs, parishes, and the District of Columbia) in the US (*N* = 3142). The data were all available from publicly available sources, and we offer an overview in Table 1. The dataset included all the counties or county equivalents for which information was available.

**Table 1:** Overview of Study Variables

Variable (Weight)	Description	Min.	Max.	***M***	***SD***
**Mask Wearing**		.26	.99	.72	.13
**Health behaviors (30%)**					
Smokers (10%)	Percent county adults who smoke.	5.91	41.49	17.47	3.61
Obesity (5%)	Percent county adults who are obese.	12.40	57.70	32.86	5.45
Food environment (2%)	USDA Food environment index (reverse scored)	0.00	10.00	2.55	1.15
Inactive (2%)	Percent county residents who are physically inactive.	9.50	49.90	27.37	5.70
No Access (1%)	Percent of county residents without access to physical activity.	0.00	100.00	62.81	23.37
Excessive drinking (2.5%)	Percent of county adults who binge or heavy drink.	7.81	28.62	17.51	3.15
Alcohol driving (2.5%)	Percent of driving deaths involving alcohol.	0.00	100.00	28.33	15.02
STI (2.5%)	Percent of residents with a sexually transmitted infection.	35.80	6120.30	401.07	283.02
Teen births (2.5%)	Number of births per 1000 females age 15–19.	2.00	103.00	29.89	14.21
**Clinical Care (20%)**					
Uninsured (5%)	Percent of county residents under age 65 without health insurance.	2.26	33.75	11.48	5.14
Primary care (3%)	Ratio of county residents to primary care physicians.	3.67	46,784.00	2613.83	2386.57
Dentists (1%)	Ratio of county residents to dentists.	3.67	29,650.00	2887.22	2445.21
Mental health (1%)	Ratio of county residents to mental health providers.	3.67	24,265.00	1737.74	2357.78
Preventable hospital (5%)	Hospital stays for ambulatory sensitive conditions per 100,000 county residents who are Medicare enrollees.	536.00	16,851.00	4858.53	1841.55
Flu (2.5%)	Percent of Medicare enrollees who did not get a flu shot.	34.00	96.00	58.26	9.77
Mammogram (2.5%)	Percent of women age 65 to 74 who did not receive mammogram screening.	35.00	87.00	59.34	7.67
**Social and Economic Factors (40%)**					
High school (5%)	Percent of county adults not graduating high school.	0.00	74.36	11.26	7.17
Some college (5%)	Percent of county residents age 25–44 without some college education	0.00	84.82	42.11	11.83
Unemployed (10%)	Percent county residents age 16 or older unemployed and seeking work.	1.30	19.90	4.13	1.50
Child poverty (10%)	Percent of county residents under age 18 living in poverty.	2.50	68.30	21.11	8.90
Single parent (5%)	Percent of children in the county who live in a house headed by a single parent.	0.00	87.00	32.36	10.65
Violent crime (2.5%)	Number of violent crime offenses per 100,000 county residents.	0.00	1819.51	251.91	192.51
Injury (2.5%)	Number of deaths due to injury per 100,000 county residents.	22.00	320.00	86.91	25.82
**Physical environment (10%)**					
Air pollution (2.5%)	Average daily density of fine particle matter in PM2.5	3.00	19.70	9.02	1.97
Water pollution (2.5%)	Presence of water violations.	0.00	1.00	0.37	0.48
Severe housing (2%)	Percent of households that are either overcrowded, have high housing cost, lack a kitchen or lack plumbing.	3.22	70.89	13.87	4.58
Drive alone (2%)	Percent of county residents who drive alone to work.	5.00	96.00	79.64	7.68
Long commute (1%)	Percent of county residents who drive at least 30 min to work.	0.00	83.00	31.46	12.52
**Controls**					
65-plus	Percent of county residents age 65 or older.	4.83	57.59	19.27	4.71
Female	Percent of county residents who are women.	26.84	56.87	49.89	2.28
White	Percent of county residents who are non-Hispanic White.	2.69	97.89	75.99	20.19
Rural	Percent of county residents who live in a rural area.	0.00	100.00	58.58	31.48
Democrat	Percent of county residents who voted Democrat in the 2016 election.	0.03	0.96	0.33	0.16

#### Mask wearing

Data concerning *Mask Wearing* came from the *New York Times* dataset [33] available on GitHub (https://github.com/nytimes/covid-19-data/blob/master/mask-use/README.md). Specifically, the *Times* and Dynata conducted interviews with 250,000 from July 2 to July 14, 2020. Participants were asked, “How often do you wear a mask in public when you expect to be within six feet of another person,” and response options included *never*, *rarely*, *sometimes*, *frequently*, and *always*. The researchers then aggregated to the county level by weighting the responses by age and gender of the participant, and using the participant’s zip code to identify the location. Thus, the data represent how likely people in a given county were to wear a mask in July 2020. For the current study, we used the percent of county residents who reported *frequently* or *always* wearing their mask as the dependent variable.

#### Health factors

All health factors data were collected from the County Health Rankings & Roadmaps website (https://www.countyhealthrankings.org). The site provides detailed estimates for US counties, with all data publicly available.

Nine variables were used to assess *Health Behaviors*. These included the percent of county residents who smoked, were obese, were physically inactive, had no access to physical activity, had a sexually transmitted infection, and engaged in excessive drinking, as well as the food environment index, the rate of alcohol-related driving deaths, and teen birth rate.

Seven variables were used to reflect *Clinical Care*. These included the percent of county residents who were uninsured, did not receive mammogram screening, and did not obtain a flu shot, as well as the rate of primary care physicians, dentists, and mental health providers relative to the population, and finally, the rate of preventable hospital stays.

Eight variables were included as *Social and Economic Factors*. These included the violent crime rate and injury death rate, as well as the percent of children living in poverty and the percent of single-parent households, and the percent of county residents without some college, with no high school degree, and who were unemployed.

The *Physical Environment* was measured with five items: air pollution, the presence of water pollution violations, and the percent of county residents who experience severe housing, commute alone, and have long commutes.

Consistent with previous scholars [25, 34], we computed each broader health factor by summing the standardized and weighted variables subsumed under that measure (see Table 1 for an overview of the weights). As evidenced in our theoretical framework and hypotheses, we were interested in the health-focused culture in each county. However, the data in the County Health Rankings & Roadmaps largely represent health deficits, such as the percent of residents who smoke. Thus, we multiplied each final health factor score by − 1 such that higher scores were then reflective of greater health focus.

### Controls

We controlled for a number of factors that could potentially influence *Mask Wearing*. Women, older individuals, and those living in suburban or urban areas are more likely to wear masks than were their peers [14]. Therefore, in drawing from data available from the County Health Rankings & Roadmaps website, we controlled for the percent of county residents who were age 65 or older (*65-plus*), *Female*, and living in rural areas (*Rural*). Race and racism are related to COVID-19 risk and mortality [35], so we controlled for the percent of county residents who were *White*. Finally, political persuasion is related to mask wearing [17]. As such, we controlled for the percent of county voters who voted for Hillary Clinton (a Democrat) in the 2016 presidential election (*Democrat*). The Guardian made these data available on GitHub (https://github.com/tonmcg/US_County_Level_Election_Results_08-20), and others have used the data to examine social attitudes and behaviors [36].

### Empirical approach

Means, standard deviations, and bivariate correlations were computed for all variables. The data are not independent, as counties are nested within states, and state-level effects concerning health and COVID-19 restrictions could influence the results [37]. As a result, common estimation techniques, such as ordinary least squares regression, could result in misestimation and Type I error inflation [38]. To accommodate for these possibilities, we used two-level random effects regression models to test the hypotheses. We estimated the model using restricted maximum likelihood estimation [39]. The state code was included as a random effect variable, and all other variables were first standardized and then specified as fixed effects. Finally, we report intraclass correlations, Akaike’s Information Criterion (AIC), and Schwarz’s Nayesian Criterion (BIC).

## Results

### Descriptive statistics

**Fig. 1:**
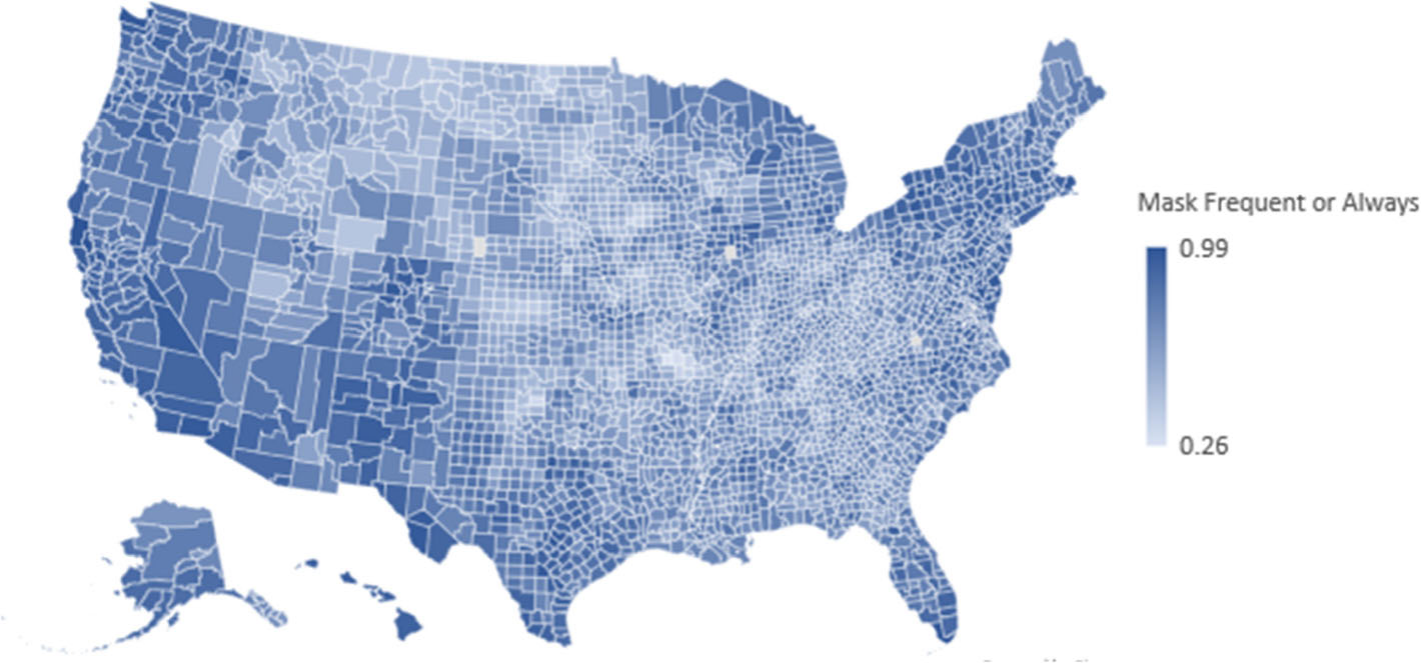
Representation of county residents who report frequently or always wearing a mask

As seen in Table 1, most (72%) county residents reported wearing a mask in public frequently or always. There was considerable variability, with a range of .26 to .99. The heterogeneity is also illustrated in Fig. 1, where we plot county-level mask wearing on a map of the US. Table 2 provides the bivariate correlations among the controls, health factors, and mask wearing. *Mask Wearing* was most prevalent in counties (a) with fewer White residents, (b) with fewer rural residents, (c) that lean Democrat in their political orientation, (d) where residents engage in healthy behaviors, and (e) that have a less healthy physical environment.

**Table 2:** Bivariate correlations among study variables

Variable	1	2	3	4	5	6	7	8	9	10
1. 65-plus	–									
2. Female	.078	–								
3. White	.392	.028	–							
4. Rural	.484	−.192	.313	–						
5. Democrat	−.262	.168	−.555	−.47	–					
6. Health behaviors	.145	.001	.228	−.207	.051	–				
7. Clinical care	.022	.121	.317	−.365	.300	.470	–			
8. Social and economic factors	−.124	.003	.449	−.147	−.137	.669	.508	–		
9. Physical environment	.324	−.208	.260	.301	−.257	.215	.130	.261	–	
10. Mask wearing	−.144	.073	−.357	−.375	.503	.252	.178	−.034	−.338	–

Notes. *r* ≥ ± .05, *p* < .05

### Hypothesis testing

Results of the two-level random intercept model with health factors predicting mask wearing are shown in Table 3. Focusing first on the unconditional model, the county-level (Level 1) and state-level (Level 2) differences in intercepts were both statistically significant; thus, the percent of county-residents who frequently or always wore masks varied based on the state and the counties within the state. The intraclass correlation (ρ = .60) shows the appropriateness of including the state in the model as a random second-level variable.

**Table 3:** Results of Two-Level Random Intercept Model with Health Factors Predicting Mask Wearing

Variable	Unconditional Model	Full Model
Fixed Effects		
Intercept	.747*** (.016)	.676*** (.049)
Health behaviors		.120*** (.022)
Clinical care		.042 (.025)
Social and economic factors		.023 (.014)
Physical environment		−.230*** (.047)
Covariates		
65-plus		.001 (.003)
Female		−.004 (.002)
White		.008* (.004)
Rural		−.005 (.003)
Democrat		.051*** (.003)
Variance components		
Level 1 – County-level (σ^2^)	.008*** (.000)	.006*** (.000)
Level 2 – State-level (τ_00_)	.012*** (.002)	.006*** (.001)
AIC	− 6046.07	− 5434.13
BIC	− 6033.97	− 5422.50

Notes. *p < .05. **p < .01. ***p < .001. Standard errors presented in parentheses

We hypothesized that as the four health factors – *Health Behaviors, Clinical Care, Social and Economic Factors,* and *Physical Environment* – increased, so too would the percent of county residents who frequently or always wore masks. As seen in Table 2, *Health Behaviors* held a significant, positive association with *Mask Wearing* (estimate = .120, standard error = .021, *p* < .001); thus, Hypothesis 1 was supported. Neither *Clinical Care* (estimate = .042, standard error = .025, *p* = .09) nor *Social and Economic Factors* (estimate = .023, standard error = .014, *p* = .10) held a significant association with *Mask Wearing*, so Hypotheses 2 and 3 were rejected. Finally, *Physical Environment* was significantly associated with mask wearing, but in the opposite direction to what was predicted (estimate = −.230, standard error = .047, *p* < .001); therefore, Hypothesis 4 was rejected.

### Supplemental analyses

We computed a number of supplemental analyses to examine the robustness of the results. First, based on previous scholarship in the area [25, 34], we weighted health factors. It is possible that unweighted health factors might offer a different pattern of findings when examining other health-related behaviors. We examined as much through a two-level random effects model with unweighted predictors. Results show that the model fit with the unweighted variables (AIC = − 6740.98, BIC = − 6728.88) was worse than the unconditional model and the full model. Thus, we did not interpret the findings.

In addition, it is possible that because *Mask Wearing* is a behavioral choice, individual health behaviors are the best predictors to include in the analyses. We tested this possibility, including the controls and the nine *Health Behaviors* described in the Methods in the analysis. Results showed that the model fit (AIC = − 6285.40, BIC = − 6273.45) was worse than the unconditional model and the full model. Thus, we did not interpret the findings.

## Discussion

The purpose of this study was to examine the manner in which health determinants at the county level were predictive of mask wearing. We framed our study in institutional theory [18, 19], arguing that counties represent geographic communities with shared interests, resources, and understandings around health. Results showed that as of July 2020, on average, 72% of county residents reported wearing a mask frequently or all of the time when outdoors and within 6 ft of another person. However, there was considerable variability (see Fig. 1), and thereby demonstrating the value of considering health determinants as a way to tease out the nature of the differences. People were more likely to wear masks when residing in counties where (a) healthy behaviors were commonplace and (b) the health-related physical environment was poor. The patterns remained even when considering county demographics and political leanings of the county residents. On the other hand, clinical care, and social and economic factors were not associated with mask wearing.

The importance of health behaviors suggests that counties are institutional communities where there are accepted, common practices related to promoting health [23]. In support of this position, our supplemental analyses revealed that exclusively focusing on individual health behaviors (e.g., smoking, excessive drinking, physical inactivity) resulted in a poor fitting model. Thus, it is the collective behaviors—more so than the individual determinants—that are predictive of mask wearing. In this way, the health behaviors represent taken-for-granted, commonly understood social behaviors that are bound within a particular institutional environment [18].

Interestingly, the health-related physical environment was negatively associated with mask wearing. Thus, people who lived in counties marked by pollution, severe housing, and long commutes likely to be taken alone, are all more likely to wear a mask than their colleagues. It is possible that, given the pollution and dense population, people in these counties are acutely aware of the need for and value of wearing a mask. From a different perspective, the health-related physical environment held a positive association with the percent of county residents living in rural settings and a negative association with the percent of county residents who voted Democrat. Previous researchers have also shown that rural setting [14] and voting patterns [17] are linked with mask wearing.

The study was also marked by what we *did not* find: an association between mask wearing and either social and economic factors or clinical care. Previous researchers have observed that social and economic factors were reliably predictive of health outcomes [25]. Others have found that social factors, such as racism [35], and clinical care factors, including the primary care physician rate [32], were predictive of COVID-19 outcomes. These relationships did not materialize in our study, though. Instead, health behaviors were most predictive of mask wearing. It is also possible a similar pattern would emerge for other steps aimed at preventing COVID-19 spread, such as staying six feet from others, avoiding crowds, hand washing, and daily health monitoring, all of which are recommended by the Centers for Disease Control and Prevention.

### Contributions, implications, limitations, and future directions

There are many contributions and implications from the current study. From a research perspective, our study adds to the growing body of scholarship showing that county-level factors can influence health, health behaviors, and other community-level outcomes. For example, the relationship between pollution and poor health is offset in counties where people have a chance to be physically active [40], and racial biases at the county level are predictive of health disparities [41, 42], the use of lethal force by police [43], Black school children’s punishments [44], and sports fans’ reactions to Black Lives Matter protests [45]. Our study contributes to this burgeoning line of inquiry by showing that health determinants are predictive of county residents’ mask wearing. Theoretically, we add to the small but growing body of scholarship showing the ways in which institutional thinking can help make sense of and explain health outcomes in communities [24].

Our research draws attention to the importance of institutional infrastructures when considering health outcomes [46]. Institutional infrastructures, which are distinct from physical elements of infrastructure, entail the underlying assumptions, attitudes, and structures within a given setting [46]. Whereas research of institutional infrastructures is largely tied to understanding governance systems within organizational fields [46], we point to impact of institutional infrastructures regarding individual behaviors, specifically health-related behaviors. Considering our research offered mixed results of the influence of institutional infrastructures, future research should seek to clarify the precise tenets of institutional infrastructure that are most likely to influence human behavior, especially related to health outcomes.

Though the study has many strengths and implications, we also note potential limitations. First, our dependent variable was the county residents’ self-report mask wearing behavior at a single point in time. Though intentions are a strong predictor of health behavior [47], we do not know if the respondents actually wore the masks frequently or always. Second, our dataset allowed for examination of counties around the United States, but we also recognize that county-level estimates do not capture potential within-county variability. Related to this point, the study findings might not be applicable to settings outside the United States. Finally, our focus was on health determinants, while also considering county demographics and voting. The nature of the data meant that we did not consider psychological factors that might influence mask wearing, such as prosociality [48], altruism [49], or related constructs.

Finally, we see several opportunities for future directions. First, we noted the small but growing body of scholarship examining health outcomes at the county levels. Additional research in this area is warranted. Second, in our implications, we noted potential levers public health officials could use to create change and healthier counties. This is not a new topic, but it nevertheless warrants further analysis and research among public health scholars. Finally, we focused on mask wearing – an important behavior helping to stop the spread of COVID-19 [7, 12]. At the time of this writing, the medical community had just begun to vaccinate people in high-risk categories. As the data become available, future researchers would likely find value in exploring the ways in which health determinants predict vaccination rates and people’s desire to receive the vaccination.

## Data Availability

All relevant data are available at 10.6084/m9.figshare.14711067.v1.
